# Developing and Validating a Lung Cancer Risk Prediction Model: A Nationwide Population-Based Study

**DOI:** 10.3390/cancers15020487

**Published:** 2023-01-12

**Authors:** Katrine H. Rubin, Peter F. Haastrup, Anne Nicolaisen, Sören Möller, Sonja Wehberg, Sanne Rasmussen, Kirubakaran Balasubramaniam, Jens Søndergaard, Dorte E. Jarbøl

**Affiliations:** 1 OPEN—Open Patient Data Explorative Network, Odense University Hospital, 5000 Odense, Denmark; 2 Research Unit OPEN, Department of Clinical Research, University of Southern Denmark, 5230 Odense, Denmark; 3 Research Unit of General Practice, Department of Public Health, University of Southern Denmark, 5000 Odense, Denmark

**Keywords:** cancer diagnosis, automated risk calculation, prediction models, register data, lung cancer, socioeconomic status

## Abstract

**Simple Summary:**

We aimed to predict the individual one-year risk of lung cancer for all individuals aged 40 or above living in Denmark on 1 January 2017. For prediction, we used a wide range of data from nationwide Danish databases on health and sociodemographics from 2007–2016. We developed separate models for individuals with or without previous cancer diagnosis within ten years. Half of the data was used for the development of the prediction models, and the other half for evaluating the resulting models. In the population without and with previous cancer, 4274 (0.15%) or 482 (0.3%) individuals received a lung cancer diagnosis in 2017. For both populations, older age was a relevant predictor, and the most complex models, containing variables related to diagnoses, medication, general practitioner, and specialist contacts, as well as baseline sociodemographic characteristics, performed best. Our prediction models demonstrated a potential to support identifying individuals at risk of lung cancer.

**Abstract:**

Lung cancer can be challenging to diagnose in the early stages, where treatment options are optimal. We aimed to develop 1-year prediction models for the individual risk of incident lung cancer for all individuals aged 40 or above living in Denmark on 1 January 2017. The study was conducted using population-based registers on health and sociodemographics from 2007–2016. We applied backward selection on all variables by logistic regression to develop a risk model for lung cancer and applied the models to the validation cohort, calculated receiver-operating characteristic curves, and estimated the corresponding areas under the curve (AUC). In the populations without and with previously confirmed cancer, 4274/2,826,249 (0.15%) and 482/172,513 (0.3%) individuals received a lung cancer diagnosis in 2017, respectively. For both populations, older age was a relevant predictor, and the most complex models, containing variables related to diagnoses, medication, general practitioner, and specialist contacts, as well as baseline sociodemographic characteristics, had the highest AUC. These models achieved a positive predictive value (PPV) of 0.0127 (0.006) and a negative predictive value (NPV) of 0.989 (0.997) with a 1% cut-off in the population without (with) previous cancer. This corresponds to 1.2% of the screened population experiencing a positive prediction, of which 1.3% would be incident with lung cancer. We have developed and tested a prediction model with a reasonable potential to support clinicians and healthcare planners in identifying patients at risk of lung cancer.

## 1. Introduction

Lung cancer is one of the most common cancers and the leading cause of cancer-related deaths in both men and women globally [1]. The prognosis is poor because the majority of patients are diagnosed at advanced stages where curative treatment is no longer possible [2]. Therefore, early diagnosis of lung cancer is essential to improve the prognosis. However, reasons for diagnostic delays are multifactorial, challenging clinicians to identify patients in need of further investigation.

One factor is that the early stages of lung cancer are often asymptomatic. Another factor is a significant patient delay because symptoms are common and non-specific [3]. A review found that lung cancer patients have had symptoms months before diagnosis but had not assessed these symptoms as reasonable to seek medical attention [4]. Thirdly, alarm symptoms of lung cancer, such as dyspnea, loss of appetite, and prolonged cough, have a low predictive value [5] in the general population, meaning that many patients with these symptoms must be investigated to identify one with lung cancer.

Identification of high-risk patients is necessary to facilitate earlier diagnosis. To support clinicians and health administrators in this identification, other individual risk factors such as smoking [6], age and socioeconomic status [7], and previous smoking-related, breast or hematologic malignancies [8] could increase the precision in predicting individual risk of lung cancer. Risk prediction models for lung cancer have been developed. Some have focused on identifying high-risk individuals for lung cancer screening based on risk factors such as age, smoking status, occupational exposure to asbestos, pesticides or fumes, and family history of cancer [9,10]. A model by Hippisley-Cox et al., 2011 incorporated both individual risk factors and data from general practitioners’ records on presentation of alarm symptoms and systemic symptoms, e.g., tiredness, to identify patients at highest risk that should be referred for further investigation [11].

Socioeconomic status (SES) is also an important modifying factor for healthcare-seeking behaviour. Lower SES is associated with late stage cancer diagnosis [12], while low SES predicts an overall slightly worse prognosis for lung cancer patients [13]. In a recent cancer risk assessment model (CRAM) we investigated the predictive effect of combining individual-level data on risk factors, hospital and primary care visits, main medical reasons for diagnostic procedures or treatment, and SES on all types of cancer [14]. This study identified 39 and 42 predictive individual risk factors in women and men, respectively, and found that SES also affects risk prediction, but only marginally. SES may affect health outcomes for cancer survivors by influencing cancer survivors’ health information-seeking, healthcare-seeking behaviour, and cancer care decisions [15,16,17].

Denmark has a large array of high-quality national registers that provide a unique opportunity to perform large population-based studies linking information about diagnoses, medications, etc., at the individual level. Consequently, it is possible to investigate whether models based on an extensive amount of different and highly valid data can be effective methods and tools for assessing the risk of having cancer based on Danish healthcare data. As there is a possible difference in biological risk between individuals with and without prior cancer, there is a probable need for risk prediction models including both populations with and without a prior cancer diagnosis.

The objectives of this study were to develop and validate 1-year predictive models to estimate the individual risk of having lung cancer, incorporating both baseline risk factors and SES to help clinicians identify those at the highest risk for lung cancer for both individuals with no former cancer diagnosis, and in individuals with a previous cancer diagnosis. The prediction models based on already available register data could feed directly into electronic patient record systems, identify patients at increased risk of lung cancer and alert the physician to pay attention to the high-risk patients. The physician then could evaluate the individual patient by considering symptoms, family history etc.

## 2. Subjects and Methods

### 2.1. Study Design

**Figure 1:**
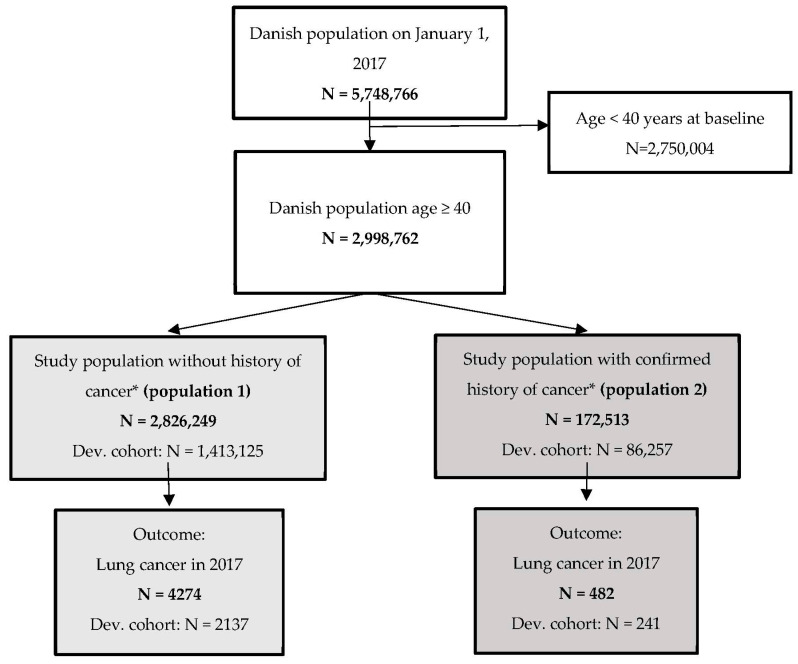
Flow chart of the study population without a history of cancer (population 1), and the study population with a history of cancer (population 2). * In the period 2007–2016.

This nationwide register-based cohort study is based on data extraction from Danish national registers covering all individuals living in Denmark on 1 January 2017, aged 40 years or above, including ten years of retrospective data (2007 to 2016). The study population was divided in a cohort of individuals without a history of confirmed cancer diagnosis from 2007 to 2016 (population 1) and a cohort of individuals with a history of cancer from 2007 to 2016 (population 2) (Figure 1). The reasons for separate models for the two populations are that the specific cancer history may have an overall effect on the pattern of contact with the health system and the patient’s health concerns. Patients with a previous cancer diagnosis may be more prone to contact their GP with symptom experiences and may have a higher attention to bodily changes due to concerns about a new cancer diagnosis compared to patients without previous cancer. In each of the two populations, our aim was to predict 1-year risk of incident of lung cancer from 1 January 2017, using the preceding 10 years of patient history as predictors.

### 2.2. Data Sources

All Danish citizens are assigned a unique civil registration number (CRN), used as the key identifier in all Danish health and social registers. The CRNs enable epidemiological studies of the entire population, currently comprising approximately 5.7 million individuals [18].

#### 2.2.1. Sociodemographic Data

The Danish Civil Registration System (CRS) includes all individuals living in Denmark [19] and maintains complete records of births, deaths, civil status, and migration status. CRS was used to identify individuals for inclusion in the study population and to obtain demographic factors, age, civil status, and ethnicity. Employment status, education, and personal income were obtained from Statistics Denmark, a national organization compiling statistical information about the Danish society for the entire population [20,21]. Civil status was categorized as either “married/cohabiting” or “living alone”. Income was ordered into tertiles; “low”, “medium”, or “high” according to disposable income after tax payment year 2016. Occupational status was categorised into “employed”, “unemployed or on welfare payment”, “education”, “early retirement”, “retirement pension”, and “unknown or missing”. Ethnicity was categorized as “Danish”, “immigrant”, “descendant (born in Denmark by parents born outside Denmark)”, and “unknown”.

#### 2.2.2. Cancer Specific Data

Data on cancer diagnoses were obtained from The Danish Cancer Registry (DCR), which contains data on all cases of cancer in the Danish population, including the date of diagnosis and tumour characteristics [22]. We used data from DCR from 2007–2016 for cancer history and from 2017 for the outcome.

#### 2.2.3. Other Health Data

Information regarding somatic and psychiatric inpatient and outpatient hospital visits and the main medical reasons for diagnostic procedures or treatment was retrieved from the Danish National Patient Register (NPR) [23]. All ICD-10 codes at level 3 relevant for conditions of interest from 2007 to 2016 were obtained. ICD-10 codes for that time frame were also used to calculate Quan’s updated version of the Charlson comorbidity index (CCI) [24].

Data on contacts with the primary healthcare system were obtained from The Danish National Health Service Register (NHSR), obtaining data on the number and types of contact and general practitioner procedures from 2007 to 2016.

Data on dispensed prescription pharmaceuticals were obtained from The Danish National Prescription Registry (DNPR) [25], containing Anatomical Therapeutic Chemical (ATC) codes, price, reimbursement, etc. We used data on all ATC-codes (level 3, e.g., A10) from 2007 to 2016.

### 2.3. Outcome (Cancer)

The primary outcome was lung cancer occurrence in 2017 extracted from DCR and dichotomized as either no or minimum of one lung cancer diagnosis in 2017 for both populations, individuals with and without a history of cancer on 1 January 2017. Exposure variables were all ICD-10 codes at level 3 from NPR as primary or secondary diagnosis, the number of contacts with a general practitioner (GP) or practicing specialist, GP procedures and measurements, and ATC codes from DNPR at level 3 (yes/no), in the exposure period 2007–2016. ATC codes had to be recorded at least twice during the exposure period to be registered as “yes”.

### 2.4. Statistical Analyses

The model development was carried out separately for population 1 and population 2. These populations were randomly split into 50% development cohorts and 50% validation cohorts, stratified by lung cancer in 2017. On the development cohort, the prediction model was constructed in three steps: (1) All level 3 ICD-10 codes and ATC codes with a prevalence below 0.1% in the cohort were excluded. (2) A backward stepwise selection applying logistic regression with a *p*-value cut-off of 0.05 was carried out separately on the remaining somatic diagnoses from NPR, somatic diagnoses from private hospitals, psychiatric diagnoses, ATC codes, and contact types in the primary sector (register data). (3) A combined backward stepwise regression with a *p*-value cut-off of 0.01 was carried out on those predictors remaining after step 2, resulting in a final prediction model with odds ratios (OR) and corresponding 95% confidence intervals reported.

For both populations, four models were conducted containing different exposure variables:

- Model A contained ICD-10 codes, ATC codes, and GP and specialist contacts, as well as age and sex
- Model B contained ICD-10 codes, ATC codes, GP and specialist contacts, and SES (including marital status and ethnicity), as well as age and sex
- Model C contained age and sex
- Model D contained SES (including marital status and ethnicity)

The resulting logistic prediction models were then applied to the validation cohort, yielding predicted cancer probabilities for each individual. Based on these, the area under the receiver operating characteristics curve (AUC under ROC) was estimated with a 95% confidence interval. 

Setting the risk cut-offs to 1% and 5%, respectively, we estimated sensitivity, specificity, positive predictive value (PPV), and negative predictive value (NPV) for models A and B in both validation and development cohorts for both populations.

## 3. Results

The cohort without a history of cancer consisted of 2,826,249 individuals, of which 4274 (0.15%) were diagnosed with lung cancer in 2017 (Figure 1). The cohort with previous cancer consisted of 172,513 individuals, of which 482 (0.3%) were diagnosed with lung cancer in 2017 (Figure 1). Characteristics of the two cohorts stratified by development and validation cohort are presented in Table 1. Overall, the lung cancer cases had a higher median age than the control group (71 and 58 years, respectively) in the cohort without a history of cancer (population 1). In the cohort with a history of cancer (population model two), the median age was 72 years for cases and 70 years for controls. In both models, cases were more often men living alone. For population 1 a lower proportion were immigrants or descendants. Cases generally had a higher income, a lower proportion of current employment, and lower education than controls, which was expected due to the higher age of the cases. In population 1 approximately 33% of cases and 1.3% of controls died during 2017, while these proportions for population 2 were about 45%, respectively 33%. As expected, due to the random splitting, no clear differences between the development and validation cohorts were observed.

### 3.1. Predictive Risk Factors for Population 1 (Individuals without a History of Cancer Diagnosis from 2007 to 2016)

In this population, the selection procedure resulted in a model (model A) including age groups, as well as 12 ICD-10 codes (only one of these, E66 obesity being protective), 14 ATC groups (including six protective predictors), and six GP consultation types and procedures (Table S3). Age was a strong predictor of lung cancer, with risk strongly increasing with age for ages 45 to 85 but decreasing after 85. Model B, including SES resulted in a similar list of diagnoses and medications but furthermore, included education groups as well as occupation and country of origin (Table S5).

### 3.2. Predictive Risk Factors for Population 2 (Individuals with a History of Cancer Diagnosis from 2007 to 2016)

In this population, the selection procedure resulted in a model (model A) including age groups, as well as 10 ICD-10 codes (only one of these, I20 Angina pectoris protective), only one ATC group (N07: Other nervous system drugs), and five GP consultation types and procedures (Table S4). Age above 60 years was a predictor of lung cancer, with no clear trend with increasing age above this threshold, all age groups above 60 years showing OR between 2 and 3. Model B, including SES resulted in a similar list of diagnoses and medications but furthermore including education groups (Table S6).

### 3.3. Validation of the Different Models, Population 1 (Individuals without a History of cancer Diagnosis within 2007 to 2016)

Model A resulted in an AUC of 0.80 (95% CI 0.79; 0.81) on the validation cohort, while model B, including SES, resulted in an only slightly higher AUC of 0.81 (95% CI 0.78; 0.82). Model C, only including age and sex, and Model D, only including SES, resulted in markedly lower AUCs of 0.75, and 0.73, respectively (Table 2).

### 3.4. Validation of the Different Models, Poulation 2 (Individuals wih a History of Cancer Diagnosis within 2007 to 2016)

Model A resulted in an AUC of 0.66 (95% CI 0.62; 0.69) on the validation cohort, while model B, including SES, resulted in an only slightly higher AUC of 0.65 (95% CI 0.62; 0.69). Model C, only including age and sex, and model D, only including SES, resulted in markedly lower AUCs of 0.58, and 0.61, respectively (Table 2).

### 3.5. Predictive Performance

**Figure 2:**
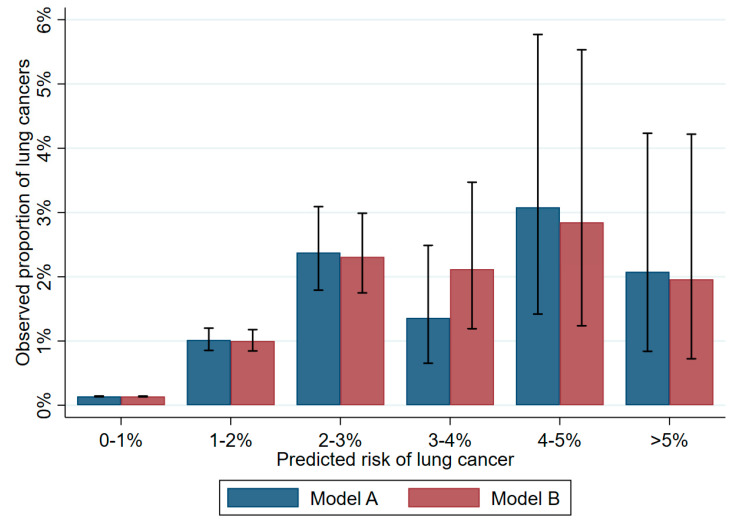
Predicted versus observed absolute risk of lung cancer (population 1).

Investigating the predictive ability of the resulting models in population 1, model A achieved a PPV of 1.3% and NPV of 98.8% with a 1% cut-off, detecting 216 true cases among 17,002 positive predictions and overlooking 1921 cases. With a 5% risk cut-off, the model achieved a PPV of 2.1% and NPV of 99.98%, detecting only seven true cases among 337 positive predictions and overlooking 2130 cases. The odds ratio for being diagnosed with cancer in 2017 was 9.33 (8.08; 10.77) with a 1% cut-off and 14.05 (5.60; 29.32) with a 5% cut-off, Table 3. Model B resulted in similar estimates (Table S1). Absolute predicted risk agreement well with observed risk for predicted risk below 3% but overestimated the risk when predicting a risk above 3% (Figure 2, Table S7). Investigating the predictive ability of the resulting models in population 2, model A achieved a PPV of 0.006 and an NPV of 0.997 with a 1% risk cut-off. Model B resulted in similar estimates (Table S2). Cut-offs above 1% were infeasible for Population 2, due to the low number of persons with high predicted risks.

## 4. Discussion

In this nationwide register-based cohort study, we developed risk prediction models for lung cancer. The best model for individuals with no previous cancer diagnosis within ten years incorporated both ICD-10 codes, ATC codes, number of GP and specialists contacts and procedures, and baseline sociodemographic characteristics. Incorporating SES only strengthened the predictive value marginally. The validation of the model resulted in a reasonable predictive value with an AUC of 0.80 in the population with no previous cancer diagnosis compared to other predictive models of lung cancer [9,10]. The absolute predicted risk agreed well with the observed risk for predicted risk below 3%. These risk cut-off scores reflected the lung cancer 1-year risk being below 1%. The algorithm can potentially be used to identify individuals who are at 9–14 times increased risk of harboring lung cancer. A recent study developing a predictive model for lung cancer using machine learning to identify clinical and laboratory data with an AUC of 0.86 was more accurate than standard eligibility criteria for lung cancer screening [26]. This study emphasized the value of using available data for identifying high-risk individuals that should be referred for further investigation, such as low-dose computed tomography [27]. Using predictive models together with the continuous improvements in imaging technology for lung cancer holds a promise of an earlier lung cancer diagnosis. A prerequisite is though, that patients contact their GP and that the GP refers them for further investigations.

For individuals with a previous cancer diagnosis, the model with the best performance had an AUC of 0.66, incorporating fewer ICD-10 codes, ATC codes, and GP and specialist contacts compared to the population with no history of cancer, implying that register data to a lesser degree predicted the risk for lung cancer in this population. For both populations, the positive predictive values with a 1% cut-off indicate that any positive results would require further follow-up to obtain a more accurate assessment of whether lung cancer was present.

### 4.1. Strengths and Limitations

The main strength of this study was the nationwide design covering the entire Danish population, including the whole population of interest, thus avoiding selection bias [28]. While data were extracted from high-quality administrative registries, the risk of information bias was minimized. That we were able to differentiate between individuals with and without previous cancer allowed for possible differences in biologic risk (e.g., genetic and epigenetic) and healthcare-seeking behaviour between the two populations.

It was a limitation of our study that we did not include information about symptom presentation, family history of cancer, or lifestyle, e.g., smoking status, and exposure to asbestos, as such are not available from the administrative registries. Tobacco smoking patterns are not a part of national registers and are frequently not registered in GPs’ patient files. Hence initiatives should be lauded, including information about lifestyle and occupational exposure to the administrative registers and addressing the relevance of the GP registering tobacco smoking habits for all patients. This would indeed improve the opportunity to stratify subjects for early detection of lung cancer. Moreover, we only had access to the outcome for 2017 and hence could not investigate changes in prediction over time.

A limitation of the statistical models applied was the use of logistic regression with backward regression and without interaction terms, hence only modelling the additive interplay between different predictors. Moreover, it is important to emphasize that we cannot claim causality between the models’ predictors and lung cancer.

A strength of this approach is though that the resulting models are easily interpretable and applicable and can be fully reported and utilized based solely on the estimates reported in this paper without the need of access to either development data or complicated model specifications. Furthermore, a strength of our approach was the employment of a separate large validation cohort.

### 4.2. Clinical Implications

It can be challenging to identify patients with an existing yet undiagnosed lung cancer because the predictive value of alarm symptoms is low, and many lung cancer patients have unspecific and common symptoms. Nowadays, the clinical guidelines for the investigation of possible lung cancer are based solely on symptoms, age, and smoking status. Our findings may be used to help inform the revision of referral guidelines to include the use of health registries.

It is our ultimate ambition that the prediction models based on already available register data could feed directly into electronic patient record systems to identify patients at increased risk of lung cancer and alert physicians. The system could integrate register data with information already available in the GP´s electronic patient record, e.g., smoking history, and consequently generate a rank-ordered list of high-risk patients for the physician to assess and prioritize patients for further evaluation for possible lung cancer. The physician then could evaluate the individual patient on a clinical basis considering symptoms, family history, and other factors not included in the prediction model.

Acknowledging that not all populations have as good registries and linking tools as in the present study, the findings may primarily be transferable to populations with comparable administrative registers. Other existing options of lung cancer identification are widely discussed, among other population-based screening. A recent UK study evaluated community-based lung cancer screening among individuals aged 55–80 years and identified from primary care records as having ever smoked showed a lower participation rate among individuals with current smoking status and socioeconomic deprivation [29] which emphasize the need for additional strategies.

As the PPV is low clinical interpretation and individualised information of risk estimates based on patient history, clinical findings [30], and risk factors such as smoking status is necessary for clinical decision-making on individual risk and management of the individual patient. Also, available register data could be combined with an algorithm incorporating presented symptoms such as hemoptysis [11]. Further, as the prediction models do not identify all lung cancer patients, clinicians cannot focus solely on high-risk patients identified by the prediction tool, and careful attention should be paid to patients with risk factors and vague symptoms of lung cancer [5]. Nevertheless, increased precision in identifying patients for further investigation will reduce unnecessary worry related to investigation for patients without increased risk of lung cancer and timely diagnosis of patients with lung cancer, ultimately improving prognosis and survival.

The first prediction model (CRAM) for identifying the individual risk of any cancer included 39 and 42 risk factors for cancer for women and men, respectively, and showed moderate accuracy. In addition to age, almost all included factors contributed statistically significantly but also only marginally to the prediction models [14]. In this study, we focused on a specific cancer type, lung cancer, and expected more precise risk estimations. However, age was still the strongest predictor of lung cancer. An ongoing challenge is diagnosing cancer with unspecific symptom presentation, and future prediction studies could focus on cancer types with unspecific symptom presentation and long diagnostic intervals [31].

## 5. Conclusions

We developed and validated prediction models to support in identifying individuals at risk of lung cancer. The register-based predictive models demonstrate a potential to support clinicians and healthcare planners in identifying individuals at risk of lung cancer that should be referred for further investigation.

## Figures and Tables

**Table 1:** Characteristics of the study populations stratified by development and validation cohorts.

	No history of Cancer (Population 1)				History of Cancer (Population 2)			
	Cases (*n* = 4274)		Controls (*n* = 2,821,975)		Cases (*n* = 482)		Controls (*n* = 172,031)	
	Development (*n* = 2137)	Validation (*n* = 2137)	Development (*n* = 1,410,988)	Validation (*n* = 1,410,987)	Development (*n* = 241)	Validation (*n* = 241)	Development (*n* = 86,016)	Validation (*n* = 86,015)
**Age** (Median (Q1–Q3))	70.9 (64.2–76.7)	71.0 (63.8–76.9)	58.3 (49.2–69.5)	58.3 (49.2–69.6)	71.7 (66.1–77.6)	72.7 (67.4–77.8)	70.2 (61.6–76.9)	70.2 (61.6–76.9)
**Age categories**								
Age 40.0–59.9	302 (14.1%)	300 (14.0%)	763,284 (54.1%)	761,932 (54.0%)	19 (7.9%)	13 (5.4%)	18,773 (21.8%)	18,831 (21.9%)
Age 60.0–79.9	1507 (70.5%)	1521 (71.2%)	537,684 (38.1%)	538,598 (38.2%)	178 (73.9%)	188 (78.0%)	52,995 (61.6%)	52,816 (61.4%)
Age ≥ 80.0	328 (15.3%)	316 (14.8%)	110,020 (7.8%)	110,457 (7.8%)	44 (18.3%)	40 (16.6%)	14,248 (16.6%)	14,368 (16.7%)
**Sex**								
Male	1089 (51.0%)	1043 (48.8%)	685,720 (48.6%)	685,644 (48.6%)	125 (51.9%)	138 (57.3%)	41,027 (47.7%)	41,128 (47.8%)
Female	1048 (49.0%)	1094 (51.2%)	725,268 (51.4%)	725,343 (51.4%)	116 (48.1%)	103 (42.7%)	44,989 (52.3%)	44,887 (52.2%)
**Marital status**								
Married	1110 (51.9%)	1139 (53.3%)	830,201 (58.8%)	831,395 (58.9%)	134 (55.6%)	127 (52.7%)	51,856 (60.3%)	51,992 (60.4%)
Widow or widower	385 (18.0%)	383 (17.9%)	131,362 (9.3%)	131,660 (9.3%)	50 (20.7%)	46 (19.1%)	14,007 (16.3%)	13,805 (16.0%)
Divorced	431 (20.2%)	418 (19.6%)	226,502 (16.1%)	225,893 (16.0%)	41 (17.0%)	49 (20.3%)	12,345 (14.4%)	12,513 (14.5%)
Unmarried	211 (9.9%)	197 (9.2%)	222,923 (15.8%)	222,039 (15.7%)	16 (6.6%)	19 (7.9%)	7808 (9.1%)	7705 (9.0%)
**Ethnicity**								
Danish	2046 (95.7%)	2032 (95.1%)	1,283,538 (91.0%)	1,283,637 (91.0%)	227 (94.2%)	236 (97.9%)	81,822 (95.1%)	81,854 (95.2%)
Immigrants/ Descendants	91 (4.3%)	105 (4.9%)	127,450 (9.0%)	127,350 (9.0%)	14 (5.8%)	5 (2.1%)	4194 (4.9%)	4161 (4.8%)
**Income** ^#^								
High tertile	877 (41.0%)	874 (40.9%)	469,621 (33.3%)	470,706 (33.4%)	94 (39.0%)	92 (38.2%)	28,703 (33.4%)	28,620 (33.3%)
Middle tertile	798 (37.3%)	774 (36.2%)	470,625 (33.4%)	469,875 (33.3%)	92 (38.2%)	91 (37.8%)	28,775 (33.5%)	28,546 (33.2%)
Low tertile/Unknown or missing	462 (21.6%)	489 (22.9%)	470,721 (33.4%)	470,390 (33.3%)	55 (22.8%)	58 (24.1%)	28,538 (33.2%)	28,848 (33.5%)
**Occupational status**								
Employed/Education	364 (17.0%)	384 (18.0%)	754,453 (53.5%)	752,975 (53.4%)	32 (13.3%)	30 (12.4%)	23,172 (26.9%)	23,256 (27.0%)
Unemployed or on welfare payment/Unknown or missing	98 (4.6%)	91 (4.3%)	101,338 (7.2%)	101,208 (7.2%)	12 (5.0%)	6 (2.5%)	3407 (4.0%)	3293 (3.8%)
Early retirement	229 (10.7%)	237 (11.1%)	120,084 (8.5%)	120,159 (8.5%)	16 (6.6%)	18 (7.5%)	6722 (7.8%)	6832 (7.9%)
Retirement pension	1446 (67.7%)	1425 (66.7%)	435,113 (30.8%)	436,645 (30.9%)	181 (75.1%)	187 (77.6%)	52,715 (61.3%)	52,634 (61.2%)
**Education**								
High education	272 (12.7%)	307 (14.4%)	416,218 (29.5%)	416,453 (29.5%)	41 (17.0%)	37 (15.4%)	22,227 (25.8%)	22,171 (25.8%)
Medium education	887 (41.5%)	850 (39.8%)	591,310 (41.9%)	591,770 (41.9%)	83 (34.4%)	99 (41.1%)	35,436 (41.2%)	35,669 (41.5%)
Low education/Unknown or missing	978 (45.8%)	980 (45.9%)	403,460 (28.6%)	402,764 (28.5%)	117 (48.5%)	105 (43.6%)	28,353 (33.0%)	28,175 (32.8%)
**Dead in year 2017**	701 (32.8%)	738 (34.5%)	18,881 (1.3%)	19,047 (1.3%)	54 (22.4%)	56 (23.2%)	6379 (7.4%)	6323 (7.4%)
**Incident lung cancer**								
Age 40.0–59.9	302 (14.1%)	300 (14.0%)			19 (7.9%)	13 (5.4%)		
Age 60.0–79.9	1507 (70.5%)	1521 (71.2%)			178 (73.9%)	188 (78.0%)		
Age ≥ 80.0	328 (15.3%)	316 (14.8%)			44 (18.3%)	40 (16.6%)		

*n*, numbers; %, percent; Q1–Q3, interquartile range; ^#^, tertiles per age category (see description in Section 2). Note: Due to confidentiality considerations, “Unknown/missing” cannot be reported separately due to low counts. Bold indicate the variable categories.

**Table 2:** Comparison of obtained AUC among different prediction models.

	Cohort without History of Cancer (Population 1)		Cohort with History of Cancer (Population 2)	
Models	Development cohort	Validation cohort	Development cohort	Validation cohort
Model A *	0.81 (0.81–0.82)	0.80 (0.79–0.81)	0.73 (0.70–0.764	0.65 (0.620–0.69)
Model B ¤	0.82 (0.81–0.83)	0.81 (0.80–0.82)	0.72 (0.68–0.75)	0.65 (0.62–0.69)
Model C #	0.75 (0.74–0.76)	0.75 (0.74–0.76)	0.58 (0.55–0.62)	0.58 (0.55–0.61)
Model D ^	0.74 (0.73–0.75)	0.73 (0.72–0.74)	0.63 (0.60–0.67)	0.61 (0.57–0.64)

AUC, area under the curve; SES, socioeconomic status (i.e., civil status, income, education level, occupation, and ethnicity). * Model A contains ICD-10 codes, ATC codes, and GP and specialist contacts, as well as age and sex. ¤ Model B contains ICD-10 codes, ATC codes, GP and specialist contacts, and SES, as well as age and sex. # Model C contains age & sex. ^ Model D contains SES.

**Table 3:** Absolute predictive performance of Model A.

Validation Cohort	Population 1 Model A		Population 2 Model A
Individuals	1,413,124		86,256
Total cancer cases	2137		241
Risk cut-off	1%	5%	1%
Number of subjects predicted above cutoff	17,002	337	2324
Cancer cases detected	216	7	14
Positive Predictive value	1.3%	2.1%	0.6%
Sensitivity	10.1%	0.3%	5.8%
Odds ratio (95% Confidence Interval)	9.33 (8.08; 10.77)	14.05 (5.60; 29.32)	2.23 (1.20; 3.84)

## Data Availability

Data supporting the findings of this study was used under a license granted specifically for the current study and therefore is not publicly available according to the data protection regulations of Danish Data Protection Agency, Statistics Denmark and the Danish Health and Medicines Authority.

## Supplementary Materials

The following supporting information can be downloaded at: https://www.mdpi.com/article/10.3390/cancers15020487/s1, Table S1: Diagnostic performance of lung cancer prediction models for absolute risk cutoffs of 1% and 5%. Cohort without history of cancer (population 1). Table S2: Diagnostic performance of lung cancer prediction models for absolute risk cutoffs of 1%. Cohort with history of cancer (population 2). Table S3: Model A: Predictive risk factors in the development cohort without history of cancer (population 1). Table S4: Model A: Predictive risk factors in the development cohort without history of cancer (population 2). Table S5: Model B; Predictive risk factors including socioeconomics in the development cohort without history of cancer (population 1). Table S6: Model B; Predictive risk factors including socioeconomics in the development cohort with cancer as outcome (population 2). Table S7: Predicted versus observed absolute risk of lung cancer (population 1 and 2)
