# Supplementary material for: Developing and Validating a Lung Cancer Risk Prediction Model: A Nationwide Population-Based Study
Source: Cancers (Basel). 2023 Jan 12;15(2):487. doi: 10.3390/cancers15020487 (PMC9856360; doi:10.3390/cancers15020487)
Supplement: Supplementary file 1 [file cancers-15-00487-s001.zip › cancers-2009691-supplementary.pdf]

Table S1. Diagnostic performance of lung cancer prediction models for absolute risk cutoffs of 1% and 5%. Cohort without history of cancer (population 1).

Model	Cohort	Cut off	Probability 0	Probability 1	Sensitivity	Specificity	PPV	NPV
Model A	Validation	1%	0.0014 (0.0013–0.0014)	0.0127 (0.0111–0.0145)	0.1011	0.9881	0.0127	0.9986
Model A	Validation	5%	0.0015 (0.0014–0.0016)	0.0208 (0.0084–0.0423)	0.0033	0.9998	0.0208	0.9985
Model B	Validation	1%	0.0014 (0.0013–0.0014)	0.0126 (0.0111–0.0144)	0.1076	0.9873	0.0126	0.9986
Model B	Validation	5%	0.0015 (0.0014–0.0016)	0.0196 (0.0072–0.0422)	0.0028	0.9998	0.0196	0.9985
Model A	Development	1%	0.0014 (0.0013–0.0014)	0.0150 (0.0132–0.0169)	0.1175	0.9883	0.0150	0.9986
Model A	Development	5%	0.0015 (0.0014–0.0016)	0.0402 (0.0216–0.0678)	0.0061	0.9998	0.0402	0.9985
Model B	Development	1%	0.0013 (0.0013–0.0014)	0.0153 (0.0136–0.0172)	0.1282	0.9875	0.0153	0.9987
Model B	Development	5%	0.0015 (0.0014–0.0016)	0.0445 (0.0239–0.0749)	0.0061	0.9998	0.0445	0.9985

Model A contains ICD-10 codes, ATC codes, and GP and specialist contacts; Model B contains ICD-10 codes, ATC codes, GP and specialist contacts, and socioeconomic status (civil status, income, education level, occupation, and country of origin); PPV, positive predictive value; NPV, negative predicted value.

Table S2. Diagnostic performance of lung cancer prediction models for absolute risk cutoffs of 1%. Cohort with history of cancer (population 2).

Model	Cohort	Cut off	Probability 0	Probability 1	Sensitiv-ity	Specificity	PPV	NPV
Model A	Validation	1%	0.0027 (0.0024–0.0031)	0.0060 (0.0033–0.0101)	0.0581	0.9731	0.0060	0.9973
Model B	Validation	1%	0.0026 (0.0023–0.0030)	0.0094 (0.0059–0.0143)	0.0913	0.9732	0.0094	0.9974
Model A	Develop-ment	1%	0.0024 (0.0020–0.0027)	0.0188 (0.0137–0.0253)	0.1784	0.9739	0.0188	0.9976
Model B	Develop-ment	1%	0.0023 (0.0020–0.0027)	0.0195 (0.0143–0.0260)	0.1867	0.9737	0.0195	0.9977

Model A contains ICD-10 codes, ATC codes, and GP and specialist contacts; Model B contains ICD-10 codes, ATC codes, GP and specialist contacts, and socioeconomic status (civil status, income, education level, occupation, and country of origin); PPV, positive predictive value; NPV, negative predicted value.

Table S3: Model A: Predictive risk factors in the development cohort without history of cancer (population 1).

Variable	OR (95% CI)*	*p*-value
**Age categories**		
Age 50–54	3.7342 (2.5571–5.4532)	<0.001
Age 55–59	8.4363 (5.9602–11.9412)	<0.001
Age 60–64	13.8833 (9.9234–19.4235)	<0.001
Age 65–69	19.4563 (13.9720–27.0931)	<0.001
Age 70–74	24.4756 (17.5997–34.0378)	<0.001
Age 75–79	23.3320 (16.6460–32.7033)	<0.001
Age 80–84	24.0146 (16.9734–33.9768)	<0.001
Age 85–89	18.2750 (12.4592–26.8056)	<0.001
Age 90–94	10.0422 (5.7733–17.4673)	<0.001
Age 95–99	9.4056 (3.3469–26.4324)	<0.001
Age +100	21.0231 (2.8737–153.7988)	0.003
**ICD-10 codes**		
D12: Benign neoplasm of colon, rectum, anus and anal canal	1.2986 (1.0966–1.5378)	0.002
D30: Benign neoplasm of urinary organs	1.7119 (1.1880–2.4669)	0.004
E66: Obesity	0.5409 (0.4142–0.7064)	<0.001
F17: Mental and behavioural disorders due to use of tobacco	2.3455 (1.9039–2.8894)	<0.001
H34: Retinal vascular occlusions	1.7964 (1.1969–2.6961)	0.005
I60: Subarachnoid haemorrhage	2.4635 (1.3873–4.3744)	0.002
I71: Aortic aneurysm and dissection	1.8653 (1.4351–2.4246)	<0.001
I73: Other peripheral vascular diseases	2.1834 (1.8353–2.5976)	<0.001
J44: Other chronic obstructive pulmonary disease	1.8479 (1.5961–2.1393)	<0.001
K90: Intestinal malabsorption	2.4953 (1.2864–4.8403)	0.007
R91: Abnormal findings on diagnostic imaging of lung	1.7304 (1.4174–2.1126)	<0.001
S62: Fracture at wrist and hand level	1.4388 (1.1730–1.7648)	<0.001
**ATC codes**		
A06: Drugs for constipation	0.7602 (0.6260–0.9231)	0.006
C05: Vasoprotectives	0.7655 (0.6494–0.9025)	0.001
C10: Lipid modifying agents	1.1590 (1.0565–1.2714)	0.002
D01: Antifungals for dermatological use	0.8072 (0.7156–0.9106)	<0.001
G03: Sex hormones and modulators of the genital system	0.7330 (0.6456–0.8323)	<0.001
J01: Antibacterials for systemic use	1.2579 (1.1236–1.4084)	<0.001
L04: Immunosuppressants	1.5111 (1.2052–1.8947)	<0.001
N02: Analgesics	1.2541 (1.1396–1.3801)	<0.001
N03: Antiepileptics	1.2975 (1.1249–1.4965)	<0.001
N07: Other nervous system drugs	1.7882 (1.5438–2.0714)	<0.001
P01: Antiprotozoals	1.2156 (1.0579–1.3968)	0.006
R01: Nasal preparations	0.7595 (0.6581–0.8766)	<0.001
R03: Drugs for obstructive airway diseases	1.6701 (1.4852–1.8781)	<0.001
R06: Antihistamines for systemic use	0.7706 (0.6649–0.8931)	0.001
**Practicing specialists (year(s) before cancer diagnoses**		
Rheumatologist (8 years)	0.8301 (0.7210–0.9558)	0.010
Gynecology/obstetrician (4 years)	0.6282 (0.4640–0.8505)	0.003
**GP consultations and procedures**		
GP C-reactive Protein testing (1 year)	1.0515 (1.0192–1.0847)	0.002
GP e-mail consultation (1 year)	0.9448 (0.9277–0.9622)	<0.001
GP spirometry (1year)	1.1501 (1.0607–1.2470)	0.001
GP spirometry with reversibility test (2 years)	1.3262 (1.0978–1.6021)	0.003
**Constant (baseline odds)**	0.000083 (0.000060–0.000115)	<0.001

Table S4. Model A: Predictive risk factors in the development cohort with history of cancer (population 2).

Variable	OR (95% CI)	*p*-value
**Age categories**		
Age 60–64	2.9134 (1.6815–5.0479)	<0.001
Age 65–69	2.9169 (1.7416–4.8855)	<0.001
Age 70–74	2.4144 (1.4446–4.0351)	0.001
Age 75–79	2.9298 (1.7348–4.9481)	<0.001
Age 80–84	2.6748 (1.5019–4.7638)	0.001
Age 85–89	2.5880 (1.3001–5.1518)	0.007
**ICD-10 codes**		
B37: Candidiasis	2.6611 (1.2794–5.5352)	0.009
I20: Angina pectoris	0.3614 (0.2008–0.6505)	0.001
I25: Chronic ischaemic heart disease	2.9072 (1.9838–4.2603)	<0.001
I73: Other peripheral vascular diseases	2.3486 (1.4644–3.7667)	<0.001
J34: Other disorders of nose and nasal sinuses	3.8564 (1.5389–9.6638)	0.004
J44: Other chronic obstructive pulmonary disease	1.9498 (1.3582–2.7990)	<0.001
K08: Other disorders of teeth and supporting structures	2.8824 (1.5738–5.2789)	0.001
K61: Abscess of anal and rectal regions	4.0095 (1.7454–9.2102)	0.001
M85: Other disorders of bone density and structure	1.9969 (1.2599–3.1651)	0.003
Q61: Cystic kidney disease	3.3455 (1.3585–8.2384)	0.009
**ATC codes**		
N07: Other nervous system drugs	1.9474 (1.2876–2.9452)	0.002
**Practicing specialists (year(s) before cancer diagnoses**		
Plastic surgery (7 years)	1.9493 (1.1983–3.1709)	0.007
Plastic surgery (10 years)	2.1116 (1.3103–3.4031)	0.002
**GP consultations and procedures**		
GP C-reactive Protein testing (5 years)	1.1334 (1.0367–1.2391)	0.006
GP e-mail consultation (1 year)	0.9293 (0.8844–0.9766)	0.004
GP Spirometry (3 years)	1.4302 (1.1944–1.7127)	<0.001
**Constant (baseline odds)**	0.000823 (0.000531–0.001278)	<0.001

Table S5. Model B; Predictive risk factors including socioeconomics in the development cohort without history of cancer (population 1).

Variables	OR (95% CI)	*p*-value
**Age categories**		
Age 50–54	3.5270 (2.4228–5.1346)	<0.001
Age 55–59	7.8246 (5.5459–11.0397)	<0.001
Age 60–64	12.1208 (8.6528–16.9788)	<0.001
Age 65–69	15.1314 (10.3937–22.0287)	<0.001
Age 70–74	18.2512 (12.3438–26.9855)	<0.001
Age 75–79	17.0994 (11.4594–25.5154)	<0.001
Age 80–84	17.3619 (11.5332–26.1362)	<0.001
Age 85–89	12.9139 (8.3080–20.0732)	<0.001
Age 90–94	6.9344 (3.8214–12.5833)	<0.001
Age 95–99	6.0882 (2.1140–17.5340)	0.001
**Female**	0.8745 (0.7947–0.9622)	0.006
**ICD-10 codes**		
D12: Benign neoplasm of colon, rectum, anus and anal canal	1.2860 (1.0859–1.5229)	0.004
D30: Benign neoplasm of urinary organs	1.6853 (1.1690–2.4297)	0.005
E66: Obesity	0.5234 (0.4009–0.6833)	<0.001
F17: Mental and behavioural disorders due to use of tobacco	2.2136 (1.7971–2.7267)	<0.001
H34: Retinal vascular occlusions	1.8386 (1.2252–2.7593)	0.003
I60: Subarachnoid haemorrhage	2.4478 (1.3791–4.3447)	0.002
I71: Aortic aneurysm and dissection	1.8971 (1.4595–2.4659)	<0.001
I73: Other peripheral vascular diseases	2.1970 (1.8526–2.6054)	<0.001
J44: Other chronic obstructive pulmonary disease	1.7586 (1.5195–2.0352)	<0.001
K90: Intestinal malabsorption	2.4741 (1.2756–4.7986)	0.007
R91: Abnormal findings on diagnostic imaging of lung	1.7273 (1.4149–2.1087)	<0.001
S62: Fracture at wrist and hand level	1.4208 (1.1582–1.7430)	0.001
**ATC codes**		
A06: Drugs for constipation	0.7428 (0.6118–0.9018)	0.003
C05: Vasoprotectives	0.7729 (0.6555–0.9112)	0.002
D01: Antifungals for dermatological use	0.8141 (0.7215–0.9187)	0.001
G03: Sex hormones and modulators of the genital system	0.7701 (0.6714–0.8834)	<0.001
J01: Antibacterials for systemic use	1.2935 (1.1551–1.4485)	<0.001
L04: Immunosuppressants	1.4100 (1.1258–1.7659)	0.003
N02: Analgesics	1.1798 (1.0717–1.2989)	0.001
N03: Antiepileptics	1.2217 (1.0586–1.4099)	0.006
N07: Other nervous system drugs	1.6868 (1.4555–1.9549)	<0.001
P01: Antiprotozoals	1.2578 (1.0942–1.4458)	0.001
R01: Nasal preparations	0.7945 (0.6883–0.9172)	0.002
R03: Drugs for obstructive airway diseases	1.6217 (1.4420–1.8238)	<0.001
R06: Antihistamines for systemic use	0.7833 (0.6757–0.9079)	0.001
**Practicing specialists (year(s) before cancer diagnoses**		
Gynecology/obstetrician (4 years)	0.6610 (0.4900–0.8917)	0.007
**GP consultations and procedures**		
GP C-reactive Protein testing (1 year)	1.0476 (1.0147–1.0814)	0.004
GP e-mail consultation (1 year)	0.9529 (0.9359–0.9701)	<0.001
GP Spirometry (1 year)	1.1361 (1.0474–1.2323)	0.002
GP Spirometry (2 years)	1.3081 (1.0813–1.5823)	0.006
**Socioeconomic status**		
Origin from not-western country	0.5253 (0.3823–0.7219)	<0.001
Medium education	0.8674 (0.7895–0.9530)	0.003
High education	0.5571 (0.4821–0.6436)	<0.001
Unemployed or welfare payment	1.6299 (1.2636–2.1025)	<0.001
Early retirement	1.4687 (1.2266–1.7586)	<0.001
Retirement	1.4478 (1.1463–1.8287)	0.002
**Constant (baseline odds)**	0.000116 (0.000083–0.000163)	<0.001

Table S6. Model B; Predictive risk factors including socioeconomics in the development cohort with cancer as outcome (population 2).

**Variables**	**OR (95% CI)**	***p*-value**
**ICD-10 codes**		
I20: Angina pectoris	0.3663 (0.2032–0.6603)	0.001
I25: Chronic ischaemic heart disease	3.0504 (2.0825–4.4680)	<0.001
I73: Other peripheral vascular diseases	2.3524 (1.4664–3.7738)	<0.001
J34: Other disorders of nose and nasal sinuses	3.9261 (1.5882–9.7051)	0.003
J39: Other diseases of upper respiratory tract	6.7511 (1.6186–28.1583)	0.009
J44: Other chronic obstructive pulmonary disease	2.0243 (1.4145–2.8969)	<0.001
K08: Other disorders of teeth and supporting structures	3.0094 (1.6499–5.4889)	<0.001
K26: Maternal care for other conditions predominantly related to pregnancy	2.9482 (1.3516–6.4308)	0.007
K61: Abscess of anal and rectal regions	3.8585 (1.6755–8.8856)	0.002
M85: Other disorders of bone density and structure	2.1665 (1.3726–3.4198)	0.001
LPRdiag_Q61	3.4777 (1.4058–8.6028)	0.007
**ATC codes**		
N07: Other nervous system drugs	1.9475 (1.2957–2.9272)	0.001
**Practicing specialists (year(s) before cancer diagnoses**		
Plastic surgery (7 years)	1.9303 (1.1813–3.1543)	0.009
Plastic surgery (10 years)	2.1614 (1.3461–3.4705)	0.001
**GP consultations and procedures**		
GP e-mail consultation (1 year)	0.9295 (0.8846–0.9767)	0.004
GP Spirometry (3 years)	1.4546 (1.2185–1.7364)	<0.001
**Socioeconomic status**		
Medium education	0.6182 (0.4651–0.8217)	0.001
High education	0.5461 (0.3804–0.7841)	0.001
**Constant (baseline odds)**	0.002728 (0.002183–0.003410)	<0.001

Table S7. Predicted versus observed absolute risk of lung cancer.

**Model**	**Cohort**	Predicted risk interval	Mean observed risk (95% CI)
		**Population 1**	
Model 1A	Development	0–1%	0.0014 (0.0013; 0.0014)
Model 1A	Development	1–2%	0.0131 (0.0112; 0.0152)
Model 1A	Development	2–3%	0.0174 (0.0125; 0.0235)
Model 1A	Development	3–4%	0.0269 (0.0163; 0.0417)
Model 1A	Development	4–5%	0.0239 (0.0097; 0.0486)
Model 1A	Development	>5%	0.0402 (0.0216; 0.0678)
Model 1B	Development	0–1%	0.0013 (0.0013; 0.0014)
Model 1B	Development	1–2%	0.0136 (0.0118; 0.0157)
Model 1B	Development	2–3%	0.0178 (0.0129; 0.0238)
Model 1B	Development	3–4%	0.0264 (0.0159; 0.0408)
Model 1B	Development	4–5%	0.0205 (0.0076; 0.0442)
Model 1B	Development	>5%	0.0445 (0.0239; 0.0749)
Model 1A	Validation	0–1%	0.0014 (0.0013; 0.0014)
Model 1A	Validation	1–2%	0.0102 (0.0085; 0.0120)
Model 1A	Validation	2–3%	0.0238 (0.0179; 0.0309)
Model 1A	Validation	3–4%	0.0136 (0.0065; 0.0249)
Model 1A	Validation	4–5%	0.0308 (0.0142; 0.0577)
Model 1A	Validation	>5%	0.0208 (0.0084; 0.0423)
Model 1B	Validation	0–1%	0.0014 (0.0013; 0.0014)
Model 1B	Validation	1–2%	0.0100 (0.0085; 0.0118)
Model 1B	Validation	2–3%	0.0231 (0.0175; 0.0299)
Model 1B	Validation	3–4%	0.0212 (0.0119; 0.0347)
Model 1B	Validation	4–5%	0.0285 (0.0124; 0.0553)
Model 1B	Validation	>5%	0.0196 (0.0072; 0.0422)
		**Population 2**	
Model 1A	Development	0–1%	0.0024 (0.0020; 0.0027)
Model 1A	Development	>1%	0.0188 (0.0137; 0.0253)
Model 1B	Development	0–1%	0.0023 (0.0020; 0.0027)
Model 1B	Development	>1%	0.0195 (0.0143; 0.0260)
Model 1A	Validation	0–1%	0.0027 (0.0024; 0.0031)
Model 1A	Validation	>1%	0.0060 (0.0033; 0.0101)
Model 1B	Validation	0–1%	0.0026 (0.0023; 0.0030)
Model 1B	Validation	>1%	0.0094 (0.0059; 0.0143)
